# Toward a positive psychology-informed framework for environmental law, human flourishing, and ecological citizenship

**DOI:** 10.3389/fpsyg.2026.1899868

**Published:** 2026-09-21

**Authors:** Wei Sijing, Wei Fanzhe

**Affiliations:** 1 College of Law and Politics, Anyang Normal University, Anyang, China; 2 School of Marxism, Xizang University, Lhasa, China

**Keywords:** ecological citizenship, environmental law, human flourishing, nature connectedness, pro-environmental behaviour, wellbeing

## Abstract

Environmental law has traditionally been framed as a regulatory mechanism for ecological protection, yet its psychological and social dimensions remain underexplored. Drawing on the theoretical foundations of positive psychology, particularly Seligman’s PERMA framework and the emerging literature on ecological wellbeing, this article proposes, as a theoretical framework rather than an established causal finding, that environmental law can function not merely as a constraint on harmful behaviour but as a structural enabler of human flourishing. We synthesise interdisciplinary evidence linking environmental legislation to positive psychological outcomes including enhanced meaning, positive emotion, social relatedness, and a sense of civic accomplishment through the lens of ecological citizenship. Environmental law, we propose, may activate motivational pathways that transform passive compliance into active engagement, thereby cultivating the psychological preconditions for ecological citizenship; this is a theoretical proposition rather than a demonstrated causal effect. We further examine the bidirectional relationship between nature connectedness, pro-environmental behaviour, and subjective wellbeing, demonstrating how robust legal frameworks can amplify these connections at a societal level. The article concludes with policy implications for designing environmental legislation that simultaneously promotes planetary sustainability and individual flourishing, proposing flourishing law paradigm as a conceptual bridge a between psychology, law, and ecology. To move beyond a purely conceptual mapping, we further develop five illustrative case analyses, each specifying a legal instrument, its hypothesised psychological mechanism, and the strength and limitations of the supporting evidence, synthesised in a comparative table.

## 1 Introduction

The relationship between law and human psychology has long been a subject of scholarly inquiry, yet the specific intersection of environmental law and positive psychological outcomes remains a nascent field of investigation. Traditional approaches to environmental law have predominantly focused on its coercive and regulatory functions the prohibition of harmful conduct, the imposition of liability, and the enforcement of conservation standards. This coercive and regulatory conception of environmental law has itself increasingly become an object of critical scrutiny in the legal literature, which has begun to interrogate the adequacy of environmental law legislation on its own terms (Baniamer and Alhendi, 2026). While these functions remain indispensable to ecological governance, they reveal only a partial picture of what environmental legislation can and does accomplish. A growing body of empirical evidence from psychology, ecology, and public health invites a reconceptualisation of environmental law as a framework that not only constrains destructive behaviour but actively nurtures the conditions for individual and collective flourishing.

Human flourishing, as conceptualised within positive psychology, encompasses the multidimensional realisation of wellbeing across emotional, relational, purposive, and civic domains (Seligman, 2011). The PERMA model comprising Positive Emotion, Engagement, Relationships, Meaning, and Accomplishment provides a theoretically robust framework for understanding how structural conditions, including legal norms and institutions, shape the psychological landscape in which people live (Butler and Kern, 2016; Goodman et al., 2018). When applied to the environmental domain, this framework opens new analytical possibilities: if contact with and stewardship of nature are demonstrably associated with psychological wellbeing (Richardson et al., 2021; Willis et al., 2024), then legal structures that protect and facilitate such contact may be understood as instruments of human flourishing.

Ecological citizenship represents the civic dimension of this relationship. Defined as the responsible pro-environmental conduct of individuals who act as agents of systemic change in both private and public spheres (Hadjichambis and Reis, 2020), ecological citizenship is simultaneously a legal, political, and psychological construct. It encompasses not merely voluntary pro-environmental behaviour but the active participation of citizens in governance processes, collective advocacy, and the cultivation of a sustainable relationship with the non-human world (Dobson, 2003; Bourban, 2023). Recent empirical work has begun to demonstrate that this civic engagement is itself a source of psychological benefit, linking ecological action to enhanced subjective wellbeing through the satisfaction of basic psychological needs (Vijaikis and Poškus, 2024).

This article proceeds from three central propositions. First, that environmental law, properly conceived, constitutes a structural determinant of human flourishing by shaping the physical, social, and normative environments in which citizens engage with the natural world. Second, that ecological citizenship, the active assumption of environmental responsibilities is a psychologically enriching practice that both requires and produces the conditions of flourishing. Third, that positive psychology provides a generative conceptual vocabulary for redesigning environmental law in ways that are simultaneously effective in ecological terms and beneficial to human psychological welfare. Together, these propositions constitute what we term the flourishing law paradigm, positive a framework that views environmental legislation not as the enemy of freedom and wellbeing, but as their structural precondition. This term was used in preference to positive law, which already carries an established and distinct meaning in legal theory, for reasons explained in Section 2.3.

### 1.1 Literature selection and synthesis approach

This article is presented as a perspective piece rather than a systematic review, and the literature underlying it was accordingly selected and synthesised narratively rather than through a pre-registered systematic protocol. Relevant work was identified through targeted searches of PsycINFO, Web of Science, Scopus, and legal databases (HeinOnline, Westlaw), using combinations of terms spanning the three fields bridged by the article: psychological (e.g., ‘PERMA’, ‘flourishing’, ‘wellbeing’, ‘positive psychology’), legal (‘environmental law’, ‘environmental legislation’, ‘environmental governance’, ‘rights of nature’), and civic-ecological (‘ecological citizenship’, ‘pro-environmental behaviour’, ‘nature connectedness’, ‘eco-anxiety’). Searches were supplemented by forward and backward citation tracking from key sources (e.g., Seligman, 2011; Dobson, 2003) and were not restricted to a fixed publication window, though the great majority of the empirical literature cited was published between 2011, following the formulation of the PERMA model, and 2026.

Table 1 summarises the databases consulted, the main search terms, the period covered, the selection criteria applied, and the method used to integrate the psychological and legal literatures.

**Table 1:** Summary of the literature search and synthesis method.

Element	Approach taken
Databases/sources consulted	PsycINFO; Web of Science; Scopus; HeinOnline; Westlaw; supplemented by forward and backward citation tracking from key anchor sources
Main search terms	Psychological: ‘PERMA’, ‘flourishing’, ‘wellbeing’, ‘positive psychology’. Legal: ‘environmental law’, ‘environmental legislation’, ‘environmental governance’, ‘rights of nature’. Civic-ecological: ‘ecological citizenship’, ‘pro-environmental behaviour’, ‘nature connectedness’, ‘eco-anxiety’. Terms were combined with Boolean AND across the three categories
Period covered	Primarily 2011 (publication of Seligman’s PERMA model) to 2026; earlier foundational sources (e.g., Wilson, 1984; Kaplan and Kaplan, 1989; Dobson, 2003) retained where conceptually foundational
Selection criteria	Peer-reviewed, English-language sources; direct relevance to the intersection of environmental law, positive psychology, or ecological citizenship; priority given to meta-analyses and systematic reviews, then empirical studies, then theoretical or doctrinal legal scholarship where empirical evidence was unavailable
Method used to integrate psychological and legal literature	Thematic mapping of categories of environmental legal instrument onto the five PERMA dimensions (Section 2.4); for each dimension, one illustrative case paired with an explicit mechanism, evidence type, and limitation (Table 2); cross-cutting synthesis of the psychological and legal literatures on institutional trust and eco-anxiety (Section 3)

The domain-by-domain organisation in Section 2.4 and the five illustrative case studies summarised in Table 2 reflect the integration method summarised in Table 1: rather than attempting exhaustive coverage of every legal instrument or psychological finding potentially relevant to each PERMA component, the article selects one analytically tractable case per domain and evaluates it against explicit criteria for mechanism and evidentiary strength (Section 2.4). This narrative and case-based selection process carries an inherent risk of selection bias, which is addressed only partially by the evidence-typing exercise in Table 2 and is discussed further as a limitation in Section 3.4.

**Table 2:** Summary of the five analytical case studies linking environmental legal instruments to PERMA domains.

Legal instrument	PERMA domain	Hypothesised mechanism	Evidence type/strength	Key limitation
Clean Air Act/EU ambient air quality directives	Positive Emotion	Emissions reduction → air quality → affect	Correlational, ecological-level	Confounded by wealth and institutional quality
Aarhus convention citizen-science mandates	Engagement	Mandated participation → flow / absorption	Correlational, more proximate link	Self-selection into participatory schemes
Bangalore community waste-management devolution	Relationships	Legal devolution → cooperative interaction → bonding	Quasi-longitudinal, single site	Limited generalisability; cultural specificity
Rights-of-nature jurisprudence (Ecuador; Whanganui River)	Meaning	Legal personhood → moral / identity recognition	Theoretical inference only	No direct pre/post psychological measurement
Urgenda Foundation v. Netherlands climate litigation	Accomplishment	Successful litigation → perceived efficacy → reduced eco-anxiety	Natural-experiment potential, untested	No corroborating survey data yet collected

To help readers distinguish the different epistemic status of claims made throughout the article, four categories are used consistently, though not marked by formal tags in the running text. Empirical evidence refers to findings reported in the peer-reviewed studies cited (e.g., meta-analytic associations, survey correlations, quasi-experimental results); it is flagged by direct attribution to a study (e.g., ‘Vijaikis and Poškus (2024) found…’) and, for the five domain-by-domain cases, by the evidence-type classification in Table 2. Theoretical assumptions are propositions imported from established frameworks positive psychology’s PERMA model, Self-Determination Theory, Attention Restoration Theory, ecological citizenship theory that are treated as background premises rather than tested within this article; these are introduced in Section 2 (‘Theoretical Foundations’) and are not themselves subject to the evidence-typing exercise in Table 2. Authors’ interpretations are inferences drawn by connecting the theoretical assumptions to the empirical evidence for example, the claim that a given legal instrument ‘exemplifies’ or ‘illustrates’ a PERMA domain in Section 2.4 is an interpretive judgement, not a finding reported in the cited source, and is generally flagged by phrasing such as ‘we interpret’, ‘in our reading’, or ‘this suggests’. Proposed hypotheses are claims put forward as testable but currently untested for example, the specific mediation pathway and moderating variables depicted in Figure 1, and the research questions listed in Section 5 and are flagged by hedged, forward-looking language (‘we hypothesise’, ‘this would predict’, ‘future research could test’). Table 2 and Figure 1 both mix these categories rather than presenting only established fact; the distinctions above are intended to make that mixture transparent rather than to claim a stronger evidentiary basis than the cited literature supports.

**Figure 1:**
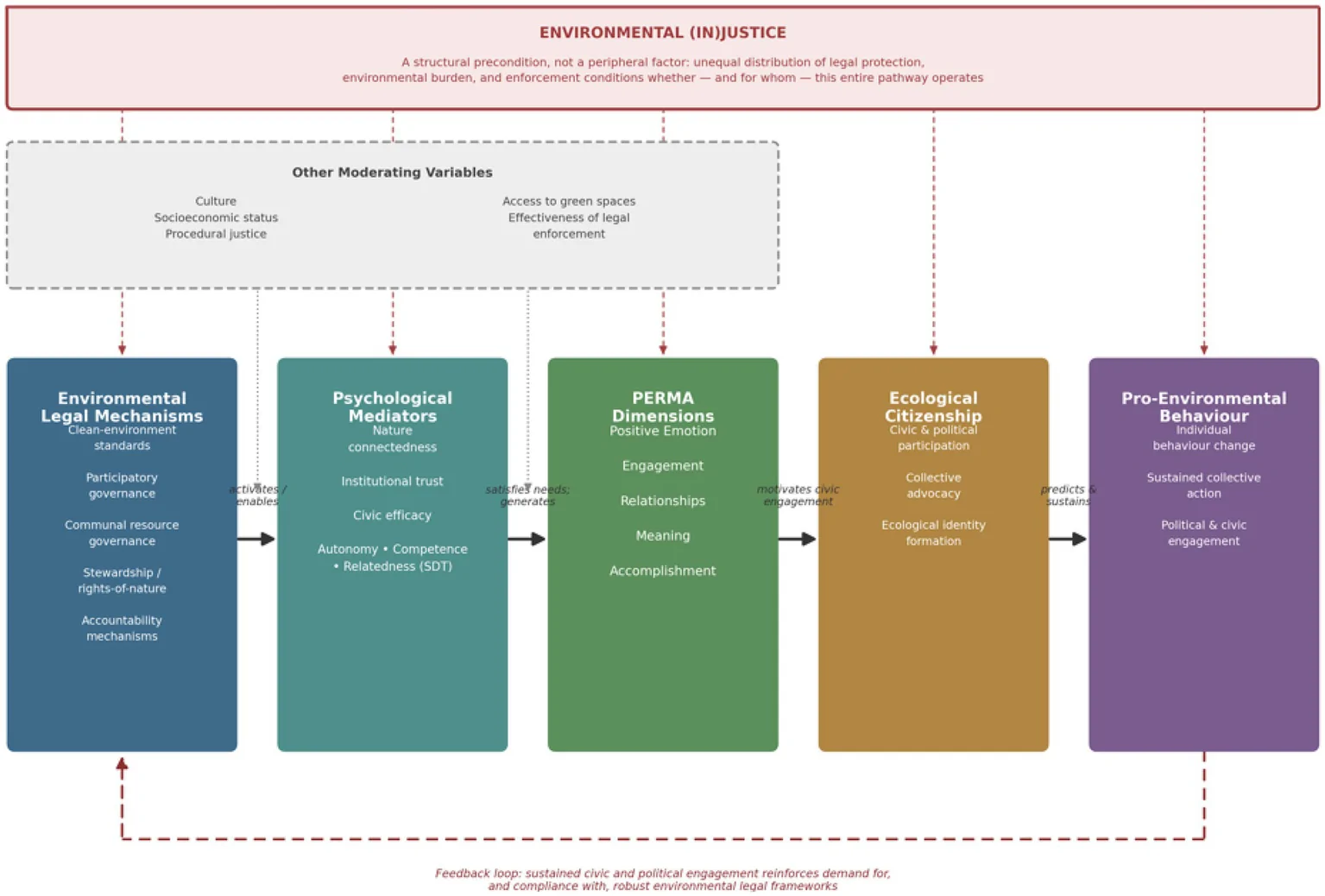
Proposed pathway from environmental legal mechanisms to pro-environmental behaviour, mediated by nature connectedness, institutional trust, civic efficacy, and autonomy, competence, and relatedness, shown as conditioned throughout by environmental (in)justice as a structural precondition, and further moderated by culture, socioeconomic status, procedural justice, access to green spaces, and the effectiveness of legal enforcement (see also Section 3 on perceived governmental adequacy and eco-anxiety), and reinforced by a civic-engagement feedback loop.

## 2 Theoretical foundations: positive psychology, environmental law, and ecological citizenship

### 2.1 Positive psychology and the PERMA framework

Positive psychology, inaugurated as a formal sub-discipline by Seligman and Csikszentmihalyi (2000), shifted the focus of psychological science from the remediation of pathology toward the understanding and cultivation of human strengths, virtues, and wellbeing. Its central contribution has been to establish wellbeing not as the mere absence of illness but as a positive state comprising multiple interrelated dimensions. Seligman’s (2011) PERMA model articulates these dimensions as Positive Emotion, Engagement (or flow), Relationships, Meaning, and Accomplishment each representing a necessary but not sufficient condition of flourishing. More recent elaborations have expanded this framework into PERMA+4, incorporating physical health, mindset, environment, and economic security as additional building blocks of wellbeing (Kern et al., 2020; Coffey et al., 2023).

The inclusion of ‘environment’ in the PERMA+4 framework is theoretically significant. It acknowledges what decades of environmental psychology have shown: that the quality of one’s physical surroundings the access to green space, the experience of natural settings, and the security afforded by a healthy ecosystem is not incidental to wellbeing but constitutive of it (Nisbet et al., 2011; Brymer et al., 2024). This insight provides the psychological foundation for the theoretical proposition developed in this article: that environmental law, by shaping the character of the physical environment, may be an important determinant of whether the environmental component of flourishing can be realised a claim examined, and only partially supported by direct evidence, in the case analyses of Section 2.4.

The inclusion of an ‘environment’ component within PERMA+4 raises a further methodological question that should be addressed directly: if wellbeing already incorporates an environmental building block, does treating environmental legal mechanisms as predictors of PERMA risk circularity, in effect measuring environmental quality’s contribution to itself? Two points limit, without eliminating, this risk. First, the analytic framework developed in this article Figure 1, and the domain-by-domain mapping in Section 2.4 and Tables 2, 3 operationalises outcomes using Seligman’s (2011) original five-component PERMA model (Positive Emotion, Engagement, Relationships, Meaning, Accomplishment), not the PERMA+4 extension; environment is not used as an outcome variable anywhere in this article’s own analysis, and the PERMA+4 discussion above is cited only to show that the wider positive psychology literature already treats environmental quality as conceptually connected to wellbeing, supporting this article’s theoretical extension rather than supplying its measurement. Second, a residual version of the concern nonetheless applies to the Positive Emotion component specifically, since the case analysed in Section 2.4.1 links clean-environment legal standards to affective wellbeing via improved environmental quality itself, placing legal mechanism and psychological outcome closer together on the same causal chain than is true for, say, Meaning or Relationships. This proximity is mitigated, though not removed, by the mediating step the model interposes between legal standard and affect (air or water quality as measured by the cited studies, rather than the law itself), and by the fact that four of the five PERMA components used as outcomes in this article’s core model are conceptually independent of environmental quality as such. Readers should nonetheless treat the Positive Emotion pathway in particular, and any future empirical test of it, with this proximity in mind.

**Table 3:** Comparative summary of legal mechanisms, psychological pathways, expected outcomes, and supporting evidence.

Legal mechanism	Psychological pathway	Expected outcome	Supporting evidence
Right to a healthy environment (constitutional/international recognition)	Moral and identity need-satisfaction; institutional legitimacy	Enhanced Meaning; reduced existential eco-anxiety	Theoretical assumption + authors’ interpretation (Atta and Sharifi, 2024); no direct pre/post psychological measurement
Public access to environmental information (Aarhus Pillar 1)	Reduced uncertainty; informed engagement	Increased Engagement; lower information-related anxiety	Empirical evidence, correlational (citizen-science/monitoring studies, Frontiers in Environmental Science, 2025)
Environmental impact assessment	Perceived procedural voice and fairness before decisions	Increased Engagement and perceived procedural justice	Theoretical extension from procedural-justice literature; not directly tested in cited studies
Citizen participation procedures (Aarhus Pillar 2)	Autonomy, competence, and relatedness need-satisfaction (SDT)	Increased Engagement and Relationships; sustained civic behaviour	Empirical evidence (Vijaikis and Poškus, 2024; SEM explaining ~41% of variance in wellbeing)
Access to environmental justice (Aarhus Pillar 3)	Perceived legal efficacy; reduced perceived governmental inadequacy	Increased Accomplishment; reduced perceived governmental inadequacy	Authors’ interpretation; proposed hypothesis (no study of justice-access psychological effects directly cited)
Climate litigation (e.g., Urgenda, Neubauer, Held)	Demonstrated institutional accountability; civic efficacy	Increased Accomplishment; eco-anxiety more likely to motivate than paralyse	Authors’ interpretation; proposed hypothesis (natural-experiment potential, untested, in (Steele, 2025)
Pollution enforcement (e.g., Clean Air Act, EU Industrial Emissions Directive)	Credibility of legal commitment; institutional trust	Positive Emotion via improved air/water quality; reduced eco-anxiety	Empirical evidence, correlational and ecological-level (Nisbet et al., 2011; Spano et al., 2021) + authors’ interpretation
Urban green-space regulations	Attention restoration; nature contact	Positive emotion; stress recovery	Empirical evidence (Nisbet et al., 2011; Doyle, 2024)

PERMA is not the only available taxonomy of wellbeing, and its selection over alternatives warrants justification. Hedonic models of subjective wellbeing (Diener’s tripartite model of life satisfaction, positive affect, and negative affect) collapse wellbeing into a single affective and cognitive index, which is well suited to large-scale survey measurement but does not distinguish the separate civic, relational, and purpose-related processes through which, this article argues, different categories of legal mechanism operate. Ryff’s six-factor model of psychological wellbeing (autonomy, environmental mastery, personal growth, positive relations, purpose in life, self-acceptance) is richer and eudaimonic in orientation, but its dimensions were not designed to align with categories of legal or institutional intervention, and its use of ‘environmental mastery’ to denote personal competence over one’s immediate surroundings risks terminological confusion in an article about environmental law specifically. Self-Determination Theory alone (autonomy, competence, relatedness), already used in this article as a set of psychological mediators (Figure 1), captures need satisfaction with precision but does not itself differentiate affective, purposive, and achievement-related outcomes as separate dimensions. Capability-based approaches to wellbeing (Sen, Nussbaum) offer a normatively rich account of justice and human functioning but lack a validated psychometric taxonomy of individual psychological states, making them better suited to development economics than to the outcome-level analysis attempted here.

PERMA was selected in preference to these alternatives for three specific reasons. First, its five components are conceptually distinct and, unlike a single hedonic index, map onto the different categories of legal mechanism identified in this article’s taxonomy: clean-environment standards onto Positive Emotion, participatory procedures onto Engagement, communal governance onto Relationships, rights recognition onto Meaning, and accountability mechanisms onto Accomplishment (Table 3). This five-to-five correspondence, rather than a general claim that law affects wellbeing, is what motivates the domain-by-domain structure of Section 2.4 and would not be available using a unidimensional or differently structured framework. Second, PERMA has a validated multidimensional psychometric instrument, the PERMA-Profiler (Butler and Kern, 2016), which distinguishes it from normative frameworks such as the capability approach and would allow the hypotheses proposed in this article to be tested empirically in future research. Third, PERMA has already been extended to PERMA+4 to incorporate physical health, environment, mindset, and economic security (Kern et al., 2020; Coffey et al., 2023), indicating that the framework is conceptually open to the kind of environmental extension this article develops rather than requiring wholesale modification. This choice is not without limitations: PERMA was not originally formulated with ecological or legal applications in mind, and its discriminant validity that is, whether its five components are empirically as separable as the model assumes has itself been questioned (Goodman et al., 2018). The application of PERMA to environmental law developed in this article should accordingly be read, consistent with the epistemic distinctions set out in Section 1.1, as a theoretical assumption adopted for its structural fit to the analytic task, not as an uncontested measurement standard.

The relationship between PERMA and Self-Determination Theory, both invoked in this article, requires clarification, since SDT appears twice: once, above, as an alternative wellbeing framework considered and set aside, and again, in Figure 1, as a component of the psychological mediators through which environmental law is proposed to operate. These uses are not inconsistent, because PERMA and SDT operate at different levels of the same explanatory chain rather than as rival descriptions of the same construct. SDT specifies a process-level mechanism: the satisfaction of three basic psychological needs autonomy, competence, and relatedness that Ryan and Deci (2000) and subsequent research treat as a proximal driver of wellbeing. PERMA, by contrast, specifies the content of flourishing itself: the five distinguishable end-states (Positive Emotion, Engagement, Relationships, Meaning, Accomplishment) that need satisfaction is theorised to help produce. On this reading, SDT explains why a given legal mechanism might promote flourishing (by satisfying autonomy, competence, or relatedness), while PERMA specifies what that flourishing consists of; this is why Figure 1 places autonomy, competence, and relatedness among the psychological mediators feeding into the PERMA dimensions, rather than treating SDT as a parallel or competing outcome measure. The terminological overlap between SDT’s relatedness and PERMA’s Relationships component in particular should not be read as redundancy: relatedness denotes the specific psychological need for social connection, whereas Relationships in PERMA denotes the broader flourishing state that need satisfaction, among other inputs, contributes to. Empirical support for this hierarchical placement rather than a side-by-side alternative comes from Vijaikis and Poškus (2024), who found that SDT need satisfaction mediated the relationship between pro-environmental behaviour and the kind of subjective wellbeing outcomes PERMA aims to capture.

### 2.2 Ecological citizenship as a psychological practice

The concept of ecological citizenship was developed most fully by Dobson (2003), who distinguished it from liberal and civic republican traditions by emphasising its orientation toward non-reciprocal obligations and its concern with ecological rather than merely political space. Where traditional citizenship is bounded by the nation-state and characterised by the receipt of rights in exchange for the discharge of duties, ecological citizenship is characterised by responsibilities that extend across species, generations, and geographic boundaries. It demands that individuals consider the ecological footprint of their actions and assume responsibilities that may not be legally mandated but are nonetheless morally binding.

This conception has been operationalised empirically by Hadjichambis and Reis (2020) and Hadjichambis and Paraskeva-Hadjichambi (2020), who define environmental citizenship as responsible pro-environmental behaviour of individuals who act and participate in society as agents of change, seeking sustainability and a healthy relationship with nature. This definition integrates the cognitive, affective, and behavioural dimensions of environmental engagement, making it amenable to psychological investigation. Critically, it positions ecological citizenship not as a passive legal status but as an active psychological and civic practice.

The psychological dimensions of ecological citizenship have received growing empirical attention. Vijaikis and Poškus (2024), drawing on Self-Determination Theory (SDT), demonstrated that pro-environmental actions constitutive of ecological citizenship satisfy the basic psychological needs of autonomy, competence, and relatedness, thereby enhancing subjective wellbeing. This finding provides a mechanism linking civic ecological action to psychological flourishing, and it implicitly suggests that legal frameworks that facilitate or mandate such actions may generate positive psychological externalities.

### 2.3 Environmental law in the flourishing law paradigm

We term this framework the flourishing law paradigm, deliberately avoiding the term ‘positive law,’ which already carries an established and distinct meaning in legal theory: classical legal positivism concerns the relationship between law and morality, and positive constitutionalism refers to state obligations to realise rights. Neither denotes the functional reorientation of environmental law developed here, and the chosen term instead signals a direct link to the human flourishing this article is concerned with: one that evaluates legislation not only in terms of its prohibitions and penalties but in terms of its capacity to enable, motivate, and sustain pro-environmental agency and psychological flourishing.

This reorientation draws on the rule of law and environmental sustainability literature. Atta and Sharifi (2024), in a systematic review, demonstrate that the rule of law is a structural precondition for environmental sustainability, noting that without enforcement of legal norms, the most rigorous ecological standards remain aspirational rather than operational. Legal frameworks thus do not merely regulate behaviour but constitute the institutional trust environment within which citizens develop ecological identities and commitments. The recent international recognition of the human right to a healthy environment (Atta and Sharifi, 2024) reinforces this understanding, situating the quality of the legal environment as a determinant of rights-realization and, by extension, of wellbeing.

Locating the originality of this paradigm relative to adjacent literatures is important, since related terms have been used for related but distinct purposes. Therapeutic jurisprudence (Wexler, 1990; Winick, 1997) examines how legal rules and procedures affect the psychological wellbeing of those who pass through legal processes, but it has developed primarily within mental health and criminal law and does not offer a domain-specific account of environmental legislation. A broader ‘law and happiness’ or wellbeing-law literature (Huang, 2010) evaluates legal rules against generalised welfare or life-satisfaction metrics without disaggregating wellbeing into the multidimensional structure that PERMA provides. Within environmental scholarship itself, ecological citizenship theory (Dobson, 2003; Hadjichambis and Reis, 2020) offers a normative-political account of the duties citizens owe to the non-human world, and the nature-connectedness literature (Richardson et al., 2021; Willis et al., 2024) documents the psychological benefits of nature contact, but neither literature systematically maps its findings onto a structured model of psychological flourishing, nor derives from that mapping specific implications for the design of legal instruments.

The originality claimed for the flourshing law paradigm developed here is therefore narrower and more specific than a general claim that environmental law affects wellbeing. It consists of three elements not, to our knowledge, previously combined in the literature: first, a systematic domain-by-domain correspondence between the five components of PERMA and distinct, identifiable categories of environmental legal instrument (clean-environment standards, participatory governance mechanisms, communal resource governance, stewardship and rights-of-nature provisions, and accountability mechanisms), rather than a single generic claim that law promotes wellbeing; second, a case-based analytic method (formalised in Section 2.4 and Table 2) that requires each proposed PERMA-law linkage to be paired with a specific instrument, an articulated mechanism, and an explicit statement of evidentiary strength and limitation; and third, an integration of the eco-anxiety and institutional-trust literature (Section 3) as a distinct empirical channel through which the credibility of legal commitments, rather than their content alone, shapes psychological outcomes. It is this combination of a structured psychological taxonomy, a disciplined case method, and an institutional-trust mechanism, rather than any single element in isolation, that we take to be the paradigm’s original contribution.

Because several existing frameworks also connect environmental governance to human welfare, it is necessary to state explicitly how the flourishing law paradigm relates to six of the most proximate: environmental justice, therapeutic jurisprudence, ecological citizenship, public health law, rights-based environmental governance, and environmental psychology. it is necessary to state explicitly how the positive law paradigm relates to five of the most proximate: environmental justice, therapeutic jurisprudence, ecological citizenship, public health law, and rights-based environmental governance. Table 4 summarises the comparison; the discussion below clarifies the evaluative question each framework asks and the question the positive law paradigm asks instead.

**Table 4:** Comparison of the flourishing law paradigm with six adjacent frameworks.

Framework	Primary orientation	Point of convergence	Key difference from the flourishing law paradigm
Environmental justice	Distributive and procedural equity in the allocation of environmental burdens and benefits across social groups	Both examine who benefits from environmental governance and how legal design shapes lived experience	Environmental justice’s evaluative currency is fairness of outcomes and processes across groups; the flourishing law paradigm’s is psychological flourishing (PERMA), largely independent of inter-group distributive comparison. The two are complementary rather than competing
Therapeutic jurisprudence	Whether legal rules, procedures, and roles have therapeutic or anti-therapeutic psychological consequences, chiefly in mental health and criminal law	Both treat law as a psychological variable rather than a purely regulatory one	Its unit of analysis is an individual’s experience of legal process (e.g., a court appearance); the flourishing law paradigm’s is the substantive content and societal-level structure of environmental legislation affecting citizens who may never enter a legal process, organised around PERMA rather than therapeutic/anti-therapeutic effect
Ecological citizenship	Normative-political theory of the duties and virtues citizens owe to non-human nature and future generations	Both treat active ecological engagement as valuable and law-relevant	Ecological citizenship theorises obligation (what citizens owe); the flourishing law paradigm theorises psychological consequence (what discharging that obligation yields for flourishing), treating ecological citizenship as one mediating practice rather than the end point of the analysis
Public health law	Legal regulation of environmental and behavioural risk factors to reduce morbidity and mortality, grounded in epidemiological outcome measures	Both examine instruments such as air and water quality regulation as determinants of population-level outcomes	Public health law’s outcomes are physical health and disease burden, typically justified on harm-prevention grounds; the flourishing law paradigm’s outcomes are positive psychological states not reducible to absence of illness, and frames the same instruments as enabling conditions for flourishing rather than only as harm controls
Rights-based environmental governance	Legal recognition of environmental entitlements as enforceable rights, held by humans or, increasingly, by non-human entities	Both draw on shared legal developments, e.g., the human right to a healthy environment and rights-of-nature jurisprudence	Rights-based governance asks a jurisprudential question standing, remedies, correlative duties; the flourishing law paradigm treats rights recognition as one mechanism among several non-rights-based mechanisms for generating flourishing, and asks an empirical question about resulting psychological experience
Environmental psychology	Empirical discipline studying how physical, built, and natural environments shape cognition, affect, and behaviour (e.g., attention restoration, stress recovery, biophilia)	Both draw on the same underlying empirical literature (e.g., Kaplan and Kaplan, 1989; Nisbet et al., 2011; Ulrich et al., 1991) linking environmental quality to psychological states	Environmental psychology is descriptive and largely apolitical, studying environment-behaviour relationships without a normative programme for legal design; it also lacks a civic or institutional dimension (ecological citizenship, institutional trust, enforcement) and does not organise its findings around a validated wellbeing taxonomy such as PERMA. The flourishing law paradigm draws on environmental psychology’s findings as evidence but adds the legal-design orientation, the PERMA-based organising taxonomy, and the civic-political mechanisms the discipline itself does not address

None of the six frameworks is superseded by the flourishing law paradigm proposed here; each addresses a related but analytically separable question: equity (environmental justice), the psychological effects of legal process (therapeutic jurisprudence), civic obligation (ecological citizenship), physical health outcomes (public health law), enforceable entitlement (rights-based governance), and environment-behaviour relationships without a legal-design programme (environmental psychology). The flourishing law paradigm’s distinctive contribution is to ask, across all these instruments, what psychological states of flourishing they generate or foreclose, using PERMA as the organising taxonomy for that question.

Table 4 above distinguishes the flourishing law paradigm from environmental justice on the grounds that the two frameworks ask different primary questions equity of distribution, in the latter case, versus psychological flourishing, in the former. That distinction should not be read as relegating justice and inequality to a secondary or optional concern within this article’s own framework. On the contrary, environmental justice is treated here as a structural precondition for the entire pathway depicted in Figure 1, rather than as one moderating variable among several: the mechanisms this article proposes clean-environment standards generating positive emotion, participatory procedures generating engagement, rights recognition generating meaning, and so on presuppose that the underlying legal protections, environmental benefits, and enforcement capacity are not themselves unequally distributed across the populations exposed to environmental harm. Where they are distributed unequally, as extensive environmental justice scholarship documents, the pathway this article describes cannot be assumed to operate uniformly, or even to operate at all, for the communities bearing disproportionate ecological burden; a legal mechanism formally available to all citizens but differentially accessible, enforced, or beneficial in practice may generate flourishing for some populations while leaving others no better off, or worse off, than before. This is why Figure 1 depicts environmental (in)justice as a condition spanning the full pathway from legal mechanism to pro-environmental behaviour, rather than as a factor affecting only the initial link between law and psychological mediators. Making this structural role explicit is intended to guard against a reading of this article’s argument that would treat legal recognition alone as sufficient for flourishing, independent of who that recognition reaches and protects in practice.

#### 2.3.1 A taxonomy of concrete legal mechanisms

The discussion so far has referred to ‘environmental law’ as a general category. To avoid treating it as an undifferentiated concept, this section makes explicit eight concrete legal mechanisms, procedures, and institutions that recur, individually, at specific points later in this article, moving from general recognition to specific procedure to enforcement outcome.

Three mechanisms establish substantive and procedural entitlements. The right to a healthy environment has been recognised at the international level by UN General Assembly Resolution 76/300 (2022) and is now codified in the constitutions or legislation of over 150 states (Atta and Sharifi, 2024), including France’s Charter for the Environment (2004) and Article 42 of Kenya’s Constitution; this mechanism is theorised to engage Meaning most directly and is discussed further in Section 3.3. Public access to environmental information is guaranteed under the first pillar of the Aarhus Convention (1998, Articles 4–5) and the EU’s Directive 2003/4/EC, and functions in the United States through the Freedom of Information Act combined with the EPA’s Toxic Release Inventory; it primarily engages Engagement. Access to environmental justice, the Aarhus Convention’s third pillar (Article 9), guarantees affected citizens and NGOs standing to challenge environmental decisions and is operationalised in the United States through citizen-suit provisions such as Clean Water Act §505 and Clean Air Act §304, which allow private parties to sue for enforcement directly; this mechanism is proposed to engage Accomplishment by making legal recourse, rather than only political advocacy, a channel for civic efficacy.

Two further mechanism’s structure participation before a decision is taken. Environmental impact assessment, required under the US National Environmental Policy Act (1970) and the EU’s EIA Directive (2011/92/EU, as amended by 2014/52/EU), obliges public and private actors to evaluate and disclose the likely environmental effects of a proposed project before it proceeds. Citizen participation procedures the Aarhus Convention’s second pillar (Article 6), NEPA’s public comment and scoping requirements, and the EU’s Public Participation Directive (2003/35/EC) require that affected citizens be consulted during that evaluation, not merely informed of its outcome. Both mechanisms are illustrated by the Aarhus citizen-science case analysed in Section 2.4.2 and are theorised to engage Engagement and, through sustained involvement, Relationships.

The remaining three mechanisms concern outcomes and enforcement. Climate litigation has produced binding judicial obligations on governments in cases such as Urgenda Foundation v. Netherlands (2019, analysed in Section 2.4.5), Neubauer v. Germany (German Federal Constitutional Court, 2021, finding the government’s climate targets insufficiently protective of future generations’ rights), and Held v. Montana (Montana Supreme Court, 2024, recognising a state constitutional right to a clean and healthful environment). Pollution enforcement mechanisms, such as the US EPA’s statutory authority to impose civil and criminal penalties under the Clean Air Act and the EU’s permitting and inspection regime under the Industrial Emissions Directive (2010/75/EU), determine whether clean-environment standards of the kind discussed in Section 2.4.1 are credible commitments or merely aspirational text, connecting directly to the effectiveness-of-enforcement moderator in Figure 1. The criminalisation of environmental pollution is itself a distinct and growing body of legal scholarship in its own right, one that examines pollution offences as a specific category of environmental crime rather than merely as an incident of regulatory non-compliance (Alhendi and Salameh, 2023). Urban green-space regulations, operationalised in jurisdictions such as the United Kingdom through statutory green belt and local planning policy, determine whether the positive-emotion benefits of nature contact discussed in Section 2.4.1 and by Doyle (2024) are realised in practice, particularly for urban populations without private access to nature.

The taxonomy above identifies categories of legal mechanism; a small number of judicial decisions illustrate how courts have given these categories interpretive content beyond the statutory text alone. The Philippine Supreme Court’s decision in Oposa v. Factoran (G.R. No. 101083, 1993) is a landmark example: the Court held that a class of children, suing on behalf of themselves and generations yet unborn, had standing to challenge timber licence agreements on the basis of the constitutional right to a balanced and healthful ecology, reasoning that this right carried an implicit obligation to preserve that ecology for succeeding generations. The decision gave early and influential judicial content to intergenerational environmental rights, a doctrine echoed decades later in the German Federal Constitutional Court’s reasoning in Neubauer discussed in Section 2.4.5. In India, the Supreme Court’s interpretation of Article 21 of the Constitution (the right to life) in a line of cases beginning with M.C. Mehta v. Union of India (1987) to encompass a right to a pollution-free environment illustrates a second judicial route to substantively similar protection: rather than relying on an express environmental rights clause, courts read environmental protection into pre-existing constitutional guarantees. Together, these examples show that the right to a healthy environment identified in the taxonomy above is not a single legal form but a family of doctrinal routes, express and interpretive, through which courts in different legal traditions have reached a similar substantive result.

A further example, illustrating that this taxonomy is not confined to European and North American legal systems, is provided by the People’s Republic of China. The 2014 amendment to the PRC Environmental Protection Law strengthened enforcement considerably, introducing uncapped daily fines for continuing violations and, under Article 58, granting qualified environmental non-governmental organisations standing to bring public interest litigation against polluters, a significant expansion of access to environmental justice within a legal system that had not previously provided for it. In 2018, the National People’s Congress amended the preamble to the PRC Constitution to include the goal of building an ‘ecological civilisation’ one of a small number of constitutional environmental provisions worldwide framed in explicitly civilisational rather than purely rights-based terms. Whether this constitutional language functions psychologically in the manner this article’s Meaning pathway (Section 2.4.4) proposes is, consistent with the epistemic distinctions set out in Section 1.1, a proposed hypothesis rather than a tested finding: no study measuring Chinese citizens’ sense of meaning before and after the 2018 amendment is known to the authors. The example is included here to illustrate the taxonomy’s applicability beyond the instruments that dominate the case analyses in Section 2.4, and to identify a further empirical opportunity for the research agenda set out in Section 5.

The mechanisms, judicial decisions, and constitutional provisions catalogued in this taxonomy vary considerably in the strength of causal evidence available to support the psychological connections proposed for them, and this variation should not be obscured by the taxonomy’s uniform presentational format. Consistent with the epistemic distinctions introduced in Section 1.1, most of the cited empirical literature establishes indirect evidence relationships between nature connectedness, psychological need satisfaction, or pro-environmental behaviour and wellbeing outcomes rather than evidence of the direct effects of environmental legislation as such. Where this article states that a given mechanism ‘engages’ or ‘is theorised to engage’ a particular PERMA domain, this denotes a theoretical proposition linking the mechanism to that domain via the indirect evidence just described, not an empirically established causal relationship between the legal instrument and psychological flourishing; Tables 2, 3 make this distinction explicit on a case-by-case basis for the mechanisms subjected to detailed analysis in Section 2.4.

### 2.4 Nature connectedness, pro-environmental behaviour, and wellbeing: the empirical evidence

#### 2.4.1 The nature connectedness–wellbeing link

The empirical literature documenting the benefits of nature contact and nature connectedness for psychological wellbeing has expanded substantially in recent years. Willis et al. (2024), in a perspective article published in Frontiers in Psychology, investigate the dynamic interplay between materialism, environmental degradation, and mental health, arguing that a societal emphasis on material acquisition contributes to an estrangement from nature with significant consequences for both mental health and environmental sustainability. They propose reintegration with nature as a fundamental response to these intertwined challenges, a conclusion that points directly to the relevance of legal frameworks governing access to natural environments.

Meta-analytic work has confirmed the reliability and magnitude of these associations. A comprehensive meta-analysis published in 2025, synthesising data from 70 cross-sectional studies conducted between 2013 and 2024 and comprising samples ranging from 102 to 4,960 participants, found robust positive relationships between nature connectedness and positive psychological outcomes, with moderating effects identified for gender, age, and the type of nature contact (Zeng et al., 2025). These findings align with earlier work by Richardson et al. (2021), who demonstrated that even relatively brief and modest encounters with natural environments reliably enhance subjective wellbeing, positive affect, and life satisfaction.

The mechanisms by which nature contact benefits wellbeing are multiple. Attention Restoration Theory (Kaplan and Kaplan, 1989) proposes that natural environments allow involuntary attention to operate freely, replenishing directed attentional resources. Stress Recovery Theory (Ulrich et al., 1991) emphasises the physiological and affective restoration afforded by natural settings. More recent psycho evolutionary accounts, such as the biophilia hypothesis (Wilson, 1984), suggest a phylogenetically deep affinity between humans and the living world that, when frustrated by urbanisation and ecological degradation, generates psychological distress. Frontiers in Psychology has published a dedicated research topic on these human-nature relationships and their implications for One Health (Brymer et al., 2024), underscoring the field’s growing scientific maturity.

#### 2.4.2 From contact to citizenship: psychological pathways

Nature connectedness does not merely benefit wellbeing passively; it also activates pro-environmental motivations and behaviours. Wild and Schulze Heuling (2024), in an empirical study of cultural and educational influences on younger generations, demonstrate that personal and social identity dimensions differentially predict pro-environmental behaviour, with identity-based engagement producing more durable and generalised effects than compliance-based approaches. This finding has important implications for legal design: legislation that cultivates ecological identity, rather than merely mandating compliance, is more likely to produce sustained behavioural change.

The mediating role of basic psychological need satisfaction is crucial here. Vijaikis and Poškus (2024), using a structural equation modelling approach with a sample of Lithuanian adolescents, found that their model could explain approximately 41% of the variance in subjective wellbeing, with pro-environmental actions satisfying autonomy, competence, and relatedness needs as the key mediating pathway. This result demonstrates that ecological citizenship is not merely instrumentally valuable for the environment but intrinsically rewarding for the citizen, suggesting that legal structures that empower ecological agency will simultaneously promote wellbeing.

The relationship between connectedness to nature and social behaviour extends beyond individual wellbeing. Isham et al. (2022), exploring the role of self-transcendent experiences as promoters of ecological wellbeing in Frontiers in Psychology, argue that experiences of awe, flow, and mindfulness often elicited by encounters with the natural world expand the sense of self in ways that increase prosocial and pro-environmental motivation. These findings suggest that environmental law, by protecting the conditions for self-transcendent natural experience, may generate psychological benefits that extend far beyond the individual to encompass relational and communal dimensions of flourishing.

### 2.5 Environmental law as an enabler of PERMA: a domain-by-domain analysis

Figure 1 summarises the conceptual model underlying the analysis that follows. It depicts the proposed pathway from environmental legal mechanisms, through six psychological mediators’ nature connectedness, institutional trust, civic efficacy, and the satisfaction of autonomy, competence, and relatedness and the PERMA dimensions these mediators activate, to ecological citizenship and, ultimately, sustained pro-environmental behaviour. The model also depicts the moderating role of institutional trust and eco-anxiety discussed in Section 3, and the feedback loop whereby civic and political engagement generated further downstream reinforces demand for, and compliance with, the legal mechanisms with which the model begins. The pathway as a whole is a proposed hypothesis rather than an established finding; the evidentiary strength of each individual link is detailed by case in Table 2, and the moderating variables are theoretical extensions not yet tested as a set (Section 3.4).

The moderating variables shown in Figure 1 are drawn from literatures already engaged elsewhere in this article and are introduced briefly here. Culture shapes the normative meaning attached to nature contact and civic duty and is one reason identity-based engagement is found to generalise across generations and settings to differing degrees (Wild and Schulze Heuling, 2024). Socioeconomic status affects the discretionary time, resources, and access to legal remedies needed to convert formal participation rights into lived engagement. Environmental inequality the uneven distribution of environmental burdens and benefits across social groups, central to the environmental justice literature discussed in Table 1 can weaken or reverse the pathway for populations that bear disproportionate ecological harm without a corresponding share of legal protection or benefit. Procedural justice, meaning the perceived fairness of participatory processes, conditions whether legal participation rights of the kind illustrated in the Aarhus Convention case (Section 2.4.2) translate into the psychological mediators the model proposes, since participation experienced as tokenistic is unlikely to satisfy autonomy or relatedness needs. Access to green spaces determines whether clean-environment standards of the kind discussed in Section 2.4.1 and by Doyle (2024) reach individuals in practice, given well-documented inequalities in the physical proximity of high-quality natural environments. Finally, the effectiveness of legal enforcement determines whether legislation functions as a credible commitment or a merely aspirational standard, consistent with the rule-of-law literature reviewed in Section 2.3 (Atta and Sharifi, 2024) and the institutional-trust findings discussed in Section 3.3. Systematic empirical testing of how each of these variables moderates the specific links depicted in Figure 1 is identified as a priority in the research agenda set out in Section 5.

To move beyond a purely conceptual mapping of PERMA onto environmental law, each of the five sub-sections below follows a common analytical procedure. For each PERMA domain, the authors identify a specific legal instrument or jurisprudential development illustrative of that domain; specify the hypothesised causal mechanism connecting the legal provision to the relevant psychological outcome. Then the authors evaluate the type and strength of the empirical evidence available correlational, quasi-experimental, or genuinely causal and note at least one plausible alternative explanation or boundary condition that qualifies the inference. This procedure does not convert a review article into an empirical study, but it disciplines the analysis by requiring that each PERMA-law linkage be traced through an identifiable mechanism and tested, where possible, against evidence of a specified type, rather than asserted by association alone. Table 2 summarises the five case analyses that result.

#### 2.5.1 Positive emotion: clean environments and affective wellbeing

The most direct pathway from environmental law to positive emotion runs through the quality of the physical environment. Laws regulating air and water quality, urban green space provision, and access to natural areas function as structural determinants of the sensory and aesthetic environment that individuals inhabit. There is strong evidence that aesthetic environmental quality is associated with positive affect, with access to green spaces, clean water, and unpolluted air reliably predicting higher levels of happiness, life satisfaction, and positive emotion (Nisbet et al., 2011; Spano et al., 2021).

The recently published Frontiers in Psychology article by Willis et al. (2024) emphasises that affluent societies’ high consumption patterns paradoxically lead to environmental degradation that undermines the very conditions of wellbeing, creating a vicious cycle of material acquisition and ecological impoverishment. Environmental law interrupts this cycle by establishing non-negotiable ecological floors—minimum standards of environmental quality that protect the positive emotional benefits of a liveable natural world. From this perspective, the Clean Air Act, the Clean Water Act, and analogous instruments in other jurisdictions are not merely public health measures but psychological welfare provisions.

An illustrative case is the tightening of fine particulate matter (PM2.5) standards under instruments such as the US Clean Air Act amendments and the EU Ambient Air Quality Directives. The hypothesised mechanism runs from legally mandated emissions reductions to measurable improvements in local air quality, to reduced physiological stress and improved mood, consistent with the affect-environmental-quality pathway identified by Nisbet et al. (2011) and Spano et al. (2021). The evidence available is largely correlational and ecological in structure: jurisdictions with stricter air-quality regulation show, on average, better air-quality indicators and higher self-reported life satisfaction, but the studies reviewed here do not isolate the legal instrument as the active causal ingredient from co-occurring economic development, healthcare access, or urban design. A plausible alternative explanation is that wealthier jurisdictions both enact stricter environmental law and enjoy higher baseline wellbeing for reasons unrelated to the law itself, such as income and institutional quality more broadly (Atta and Sharifi, 2024). The case therefore supports the plausibility of the positive-emotion pathway without establishing it as causally distinct from these confounds.

#### 2.5.2 Engagement: environmental participation and flow

Engagement, in Seligman’s model, refers to the experience of flow the state of optimal challenge and absorption that arises when one is fully immersed in a meaningful task. Law is theorised to create the conditions for engagement both directly, through the design of participatory governance mechanisms, and indirectly, through the protection of natural environments in which restorative and absorbing activities can occur.

Participatory environmental governance including environmental impact assessment processes, public consultation procedures, and citizen science initiatives provides citizens with opportunities for meaningful engagement with decisions affecting their ecological surroundings. Frontiers in Environmental Science research on nature-based citizen science initiatives demonstrates that participation in biodiversity monitoring and nature-noticing activities is associated with enhanced nature connection and wellbeing (Frontiers in Environmental Science, 2025). These activities, often supported or mandated by legal frameworks, exemplify the engagement dimension of flourishing in an ecological context.

The Aarhus Convention’s legally binding participation rights information access, public consultation, and access to justice in environmental matters offers a more tractable case for the engagement domain, because the legal instrument directly mandates the participatory mechanism rather than merely correlating with it. Where citizen-science and biodiversity-monitoring schemes are written into implementing legislation, participants report the absorption and enhanced nature connection documented in the Frontiers in Environmental Science research reviewed above (2025). This is a stronger analytical case than the positive-emotion example above, in that the legal provision (mandated participation) and the psychological outcome (engagement or flow) are more proximately linked, reducing though not eliminating the risk of confounding by unrelated socioeconomic factors. The principal limitation is selection: citizens who volunteer for participatory schemes may already possess higher baseline engagement or environmental identity, so the observed association may partly reflect self-selection into legally created opportunities rather than the legal provision generating engagement *de novo*.

#### 2.5.3 Relationships: environmental law and social capital

Environmental law’s contribution to the relational dimension of flourishing operates through multiple channels. First, shared ecological values and concerns constitute a powerful basis for social bonding. Legal frameworks that institutionalise communal environmental responsibilities through collective governance of common resources, community-based conservation, or neighbourhood green space management create structured opportunities for the development of social relationships grounded in shared ecological purpose (Anantharaman, 2014).

Second, the international and intergenerational dimensions of environmental law cultivate what Bourban (2023) calls ecological cosmopolitan citizenship a sense of solidarity and mutual obligation that extends across national boundaries and forward in time. This expanded sense of relational responsibility is associated with increased prosocial motivation and, through the satisfaction of relatedness needs, with enhanced subjective wellbeing. The human right to a healthy environment, increasingly recognised in international law (Atta and Sharifi, 2024) institutionalises these relational obligations, providing a legal basis for the claim that ecological wellbeing is an inherently social good.

Anantharaman’s (2014) study of legally structured community waste management in Bangalore offers a case in which the relational mechanism can be traced with some precision: municipal legal frameworks devolving waste-management responsibility to neighbourhood associations created formal occasions for repeated cooperative interaction among residents, which in turn generated the social bonding and shared identity associated with relatedness. Because the legal devolution preceded the observed associational activity, the temporal sequence lends the case somewhat more causal credibility than the cross-sectional associations discussed elsewhere in this article. However, the study was conducted in a single urban context among a self-selected ‘new middle class’ cohort, and the extent to which legally mandated collective resource governance produces comparable relational benefits in other institutional or cultural settings, particularly where legal mandates are imposed without local buy-in, remains untested.

#### 2.5.4 Meaning: purpose, identity, and environmental stewardship

Meaning, the sense that one’s life is oriented toward something significant and worthwhile is perhaps the most deeply psychological of PERMA’s components, and the one most intimately connected to ecological citizenship. Multiple theoretical traditions converge in emphasising the meaning-generative potential of environmental stewardship. From a virtue ethics perspective, care for the natural world is constitutive of a well-lived life (Bourban, 2022). From a psychological perspective, ecological engagement satisfies what Prentice et al. (2019) call moral needs the need to live in accordance with one’s values which are uniquely predictive of flourishing.

The law contributes to meaning making by legitimising ecological values and embedding them in the normative fabric of society. When environmental law articulates clear standards of stewardship and holds both individuals and corporations accountable to them, it signals that ecological responsibility is a serious social value, worthy of legal protection. This signal reinforces the meaning that individuals derive from their own environmental commitments, providing what might be called a normative scaffolding for ecological identity. Research by Rahe and Jansen (2023) confirms that aspects of connectedness including connectedness to nature are positively associated with flourishing, suggesting that legal structures that deepen this connection also deepen the conditions for a meaningful life.

The clearest test of the meaning pathway is provided by rights-of-nature jurisprudence, such as Ecuador’s 2008 constitutional recognition of nature’s rights and New Zealand’s 2017 grant of legal personhood to the Whanganui River. These instruments make an explicit, testable claim: that formal legal recognition of ecological entities as rights-holders reshapes the meaning citizens attach to their relationship with those entities, consistent with the moral-needs account of meaning advanced by Prentice et al. (2019). At present, however, the literature reviewed in this article contains no direct psychological measurement of citizens’ sense of meaning before and after such legal changes; the inference rests on theoretical consistency with connectedness-flourishing associations (Rahe and Jansen, 2023) rather than on evidence drawn from the rights-of-nature cases themselves. This is the weakest evidentiary case among the five and is flagged here as a priority for the empirical research agenda set out in Section 5.

#### 2.5.5 Accomplishment: civic efficacy and legal empowerment

Accomplishment refers to the sense of achievement and mastery that comes from pursuing and attaining goals. In the context of ecological citizenship, it encompasses both personal achievements (adopting sustainable practices, reducing one’s ecological footprint) and collective achievements (participating in successful advocacy, contributing to measurable improvements in environmental quality). Environmental law contributes to accomplishment primarily by creating the institutional conditions for effective action, conditions under which citizens can experience genuine efficacy in their ecological efforts.

This insight is supported by research on eco-anxiety and institutional trust. Studies have found that perceived governmental inadequacy is the strongest correlate of climate worry among higher education professionals (Steele, 2025), suggesting that ineffective environmental governance undermines not only ecological outcomes but also citizens’ sense of efficacy and agency. Conversely, strong environmental legal frameworks that demonstrably protect natural resources and hold polluters accountable provide citizens with evidence that collective action, mediated by law, can produce meaningful change. We propose that this evidence is psychologically empowering, potentially transforming ecological engagement from an exercise in futility into a genuine source of accomplishment, though the cited findings establish the underlying correlations rather than this specific transformation directly.

The 2019 Urgenda Foundation v. Netherlands ruling, in which the Dutch Supreme Court held the government legally obliged to raise its emissions-reduction targets, provides an analytically useful case for the accomplishment domain because it generated a discrete, dateable event against which shifts in perceived institutional efficacy could in principle be measured. The mechanism proposed is that successful climate litigation demonstrates that legal action can compel governmental performance, thereby directly countering the perceived governmental inadequacy identified as the strongest predictor of climate worry in the (Steele, 2025) study. Corroborating survey evidence collected specifically among Dutch citizens following the ruling is not part of the literature synthesised here, so the connection between this instrument and measured accomplishment or reduced eco-anxiety remains inferential. The case nonetheless illustrates the kind of natural experiment quasi-experimental designs bracketing a specific judicial or legislative event that the field needs in order to move from the correlational evidence reviewed throughout this article toward more defensible causal claims. Table 2 provides this.

Table 3 draws these threads together into a single comparative summary, linking each of the eight concrete legal mechanisms identified above to its proposed psychological pathway, expected outcome, and the type of supporting evidence currently available, following the same evidence-typing convention used in Table 2.

## 3 Eco-anxiety, environmental inaction, and the protective function of environmental law

### 3.1 Eco-anxiety as a psychological response to ecological crisis

The psychological costs of environmental degradation and climate change have come into sharp focus in recent years through the growing literature on eco-anxiety. Orrù and Mannarini (2024), in an article in Frontiers in Psychology, define eco-anxiety as a psychological response to the climate change emergency, encompassing emotional states of fear, worry, despair, guilt, and hopelessness related to perceived ecological threats. While eco-anxiety is not classified as a pathology, its impact on mental health, daily functioning, and quality of life is increasingly recognised as clinically significant. A meta-analysis of 25 studies published in the Journal of Environmental Psychology (2024) found a moderate negative correlation between climate anxiety and psychological wellbeing (*r* = −0.296), with effects moderated by environmental identity and mental health status. This clinical significance is underscored by recent work among university students, which finds that digital media use and environmental awareness jointly shape the eco-anxiety students experience and, through it, their broader mental health (Amjad et al., 2026).

The IPCC’s Sixth Assessment Report (2023) has confirmed the unprecedented scale of climate and ecological changes driven by human activities, and this scientific consensus has entered the public consciousness with psychological consequences. Surveys consistently show that significant proportions of populations, particularly younger generations, experience substantial climate anxiety that interferes with planning for the future, forming relationships, and pursuing meaningful goals. This burden of anxiety represents a direct and measurable cost to human flourishing, a cost that effective environmental law can, in principle, mitigate.

The account of eco-anxiety given above, and its treatment elsewhere in this article as a psychological cost that effective environmental law can help mitigate, should be balanced against an alternative reading present in the eco-anxiety literature itself. A significant body of scholarship, including work by Pihkala (2020), argues that moderate eco-anxiety is not a psychological deficit but a rational, and potentially adaptive, response to a genuine and unprecedented ecological threat, comparable to appropriate anxiety in the face of other real dangers. On this view, the aim of environmental law and policy should not be to eliminate eco-anxiety or to reassure citizens out of it, but to ensure that this anxiety is met with corresponding institutional capacity for action, so that it can be channelled into engagement rather than suppressed or pathologised. This reading complements, rather than contradicts, this article’s discussion of eco-paralysis in Section 3.2 below: the underlying concern is not anxiety itself but a mismatch between perceived threat and perceived capacity for effective response.

### 3.2 The bidirectional relationship between eco-anxiety and environmental behaviour

The relationship between eco-anxiety and pro-environmental behaviour is complex and bidirectional. While moderate levels of ecological concern can motivate protective action, excessively high levels of anxiety can produce what Albrecht (2011) calls eco-paralysis, a state of behavioural stagnation arising from feelings of helplessness. Research published in the Journal of Environmental Psychology (2024) has begun to document non-linear relationships between climate anxiety and both information-seeking and behavioural intentions, suggesting that the functional consequences of ecological distress depend critically on the perceived availability of effective responses.

This finding is deeply relevant to the present argument. Environmental law, by establishing credible commitments to ecological protection and creating institutional pathways for effective citizen action, is theorised to reduce the gap between ecological concern and effective response. When citizens believe that their actions individually and collectively and mediated by law can make a genuine difference to ecological outcomes, eco-anxiety is more likely to motivate than to paralyse. On this reasoning, the legal framework would function as a psychological resource, transforming anxiety from a barrier into a catalyst for ecological citizenship; this is a theoretical proposition extrapolated from the non-linear climate-anxiety findings above rather than a direct test of environmental law’s effect on eco-anxiety.

### 3.3 Eco-anxiety, institutional trust, and the legal environment

The relationship between eco-anxiety and institutional trust deserves particular attention. A cross-sectional mixed-methods study of 556 higher education professionals found that perceived governmental inadequacy the sense that governments are failing to take adequate action on climate change was the strongest predictor of climate worry, accounting for 26% of its variance (Steele, 2025). This finding suggests that environmental law may function not only as a direct regulator of ecological behaviour but as a symbolic expression of institutional commitment to ecological values. We propose, as a theoretical extension of this finding rather than a directly tested claim, that legal frameworks seen as credible and effective would reduce eco-anxiety by demonstrating that the institutions on which citizens depend are aligned with ecological realities.

Conversely, the enactment of strong environmental legislation can itself be a psychologically restorative act. The recognition of the human right to a healthy environment at the international level, as analysed by Atta and Sharifi (2024) signals a fundamental reorientation of legal obligations toward the protection of the ecological conditions for human flourishing. This legal development, and the institutional commitments it encodes, may provide citizens with a sense that their ecological concerns are taken seriously a recognition that mitigates existential anxiety and reinforces the meaningfulness of ecological engagement.

### 3.4 Analytical limitations and alternative explanations

A general caveat applies throughout this article and is stated explicitly here. Most of the empirical studies cited in Sections 2 and 3 establish associations between nature connectedness, wellbeing, basic psychological need satisfaction, and pro-environmental behaviour; considered individually, none of them directly demonstrates that environmental law causes human flourishing. Statements in this article suggesting that environmental law reduces eco-anxiety, activates PERMA dimensions, or restores institutional trust should accordingly be read as theoretical propositions that connect these established associations to environmental law via the mechanisms proposed in Section 2.4 and Figure 1, not as claims for which direct causal evidence is cited, unless a specific study testing that specific causal claim is named.

The case analyses developed in Section 2.4 make the PERMA-environmental law linkages more analytically tractable, but they do not resolve the central inferential problem that runs through this article: with the partial exception of the Bangalore and Urgenda cases, most of the evidence reviewed is cross-sectional and correlational, and causal direction cannot be established from the studies as designed. At least three alternative explanations deserve explicit consideration. First, reverse causality: individuals and societies with higher baseline flourishing may be more likely to demand, enact, and comply with strong environmental legislation, rather than the legislation producing the flourishing (Wray-Lake et al., 2019). Second, omitted-variable confounding: national wealth, institutional quality, and the rule of law more broadly (Atta and Sharifi, 2024) plausibly drive both stronger environmental legislation and higher subjective wellbeing independently of any specific psychological mechanism proposed here. Third, sample and publication bias: the empirical literature synthesised in Sections 2 and 3 is drawn predominantly from WEIRD (Western, Educated, Industrialised, Rich, Democratic) populations, and the meta-analytic evidence on nature connectedness (Zeng et al., 2025) itself notes moderating effects by demographic characteristics that limit generalisability to the rapidly developing and legally heterogeneous contexts in which much environmental law reform is currently occurring.

These limitations do not undermine the plausibility of the positive law paradigm, but they do mean that the paradigm is best read, at its current stage of evidentiary development, as a well-specified and empirically motivated hypothesis rather than a demonstrated causal account. The research agenda set out in Section 5, particularly the calls for longitudinal and quasi-experimental designs bracketing specific legal changes, follows directly from this analytical gap.

This article is, in its entirety, a conceptual and theoretical contribution. It does not present new empirical data, nor does it test the proposed model against any dataset; the pathway depicted in Figure 1, the domain-by-domain mapping in Section 2.4, and the taxonomy in Section 2.3 are accordingly best read as a structured set of hypotheses and conceptual propositions for future empirical investigation, not as established empirical conclusions. This point has been made piecemeal throughout the article, through the epistemic typology in Section 1.1 and the hedged language used for each proposed mechanism; it is restated here as a standing characterisation of the manuscript as a whole, so that no individual claim, however confidently worded for readability, should be mistaken for a demonstrated empirical result in the absence of a specific cited study establishing it.

A related limitation concerns geographic and cultural generalisability, which operates unevenly across the literatures this article draws on. The taxonomy of legal mechanisms in Section 2.3 has been deliberately broadened beyond European and North American instruments to include judicial and constitutional examples from the Philippines, India, and China, in part to address this concern. The underlying psychological evidence base, however, remains predominantly Western: the PERMA framework itself was developed and validated primarily in English-speaking, individualist contexts (Section 2.1), and the empirical studies on nature connectedness, self-determination, and eco-anxiety cited throughout Sections 2 and 3 draw disproportionately on North American, European, and Australian samples. This creates a mismatch the article cannot fully resolve broadening the legal examples does not, by itself, establish that the proposed psychological mechanisms operate similarly in the legal and cultural settings those examples are drawn from. Testing whether the pathway in Figure 1 holds in non-Western legal and cultural contexts, rather than merely illustrating it with non-Western legal instruments, is accordingly identified as a distinct priority for the research agenda in Section 5, separate from the general call for longitudinal and quasi-experimental designs already made in this section.

## 4 Policy implications: designing environmental law for flourishing

### 4.1 Participatory environmental governance

The evidence reviewed in this article supports the redesign of environmental law around principles of positive psychology. The most immediate implication concerns participatory governance. If ecological citizenship is psychologically enriching and if engagement with environmental decision-making satisfies basic psychological needs, then legal frameworks should be designed to maximise meaningful participation rather than reduce it to formal compliance. This means not only procedural rights to information and consultation but substantive empowerment mechanisms: community-based natural resource management, citizen science integration into regulatory processes, and environmental justice provisions that ensure equitable access to the benefits of ecological governance.

The ecological citizenship literature, particularly the work of Hadjichambis and colleagues (Hadjichambis and Reis, 2020), emphasises the importance of understanding, motivation, and capacity as the three pillars of effective ecological citizenship. Legal frameworks should be designed with all three dimensions in mind: providing citizens with the information necessary to understand ecological issues, creating incentive structures that cultivate intrinsic rather than merely extrinsic motivation, and building the institutional capacity for effective collective action.

### 4.2 Green space and environmental quality as psychological infrastructure

The evidence on nature connectedness and wellbeing supports the treatment of environmental quality and access to green space as essential psychological infrastructure. Laws governing urban planning, land use, and environmental protection should be evaluated not only in terms of their ecological outcomes but in terms of their consequences for the psychological wellbeing of urban and rural populations. The Journal of Environmental Law article by Doyle (2024) on protecting everyday nature argues that ‘everyday nature’ people’s ability to access nature nearby should be protected in law and planning policy, facilitating benefits for human health and wellbeing alongside intrinsic and extrinsic ecological advantages. This argument, which situates human wellbeing and ecological protection as complementary rather than competing values, exemplifies the flourishing law paradigm.

### 4.3 Legal frameworks for eco-anxiety mitigation

The growing evidence on eco-anxiety has direct implications for legal design. If perceived institutional inadequacy is the strongest predictor of climate worry, then the enactment of credible and effective environmental legislation is itself a public mental health intervention. Governments have obligations, under both environmental and health law, to protect their citizens from the psychological harms of ecological degradation and institutional inaction. This means not only setting ambitious climate targets but establishing transparent monitoring mechanisms, enforcement provisions, and accountability structures that allow citizens to verify institutional performance.

The flourishing law paradigm also suggests the importance of legal frameworks that empower individual ecological agency. Research consistently shows that perceived behavioural control the sense that one’s actions can make a difference is a key determinant of pro-environmental behaviour. Legal structures that recognise and validate individual contributions to collective ecological goods, such as carbon market mechanisms, green infrastructure requirements, and community energy initiatives, provide citizens with the institutional context in which efficacy and accomplishment can be genuinely experienced.

### 4.4 Educational and cultural dimensions of positive environmental law

The flourishing law paradigm extends beyond regulatory design to encompass the educational and cultural dimensions of legal governance. Environmental education, increasingly recognised in international frameworks such as the SDGs, is both a legal obligation and a psychological intervention: it cultivates the ecological identity, values, and competencies that make genuine ecological citizenship possible. Research on environmental citizenship among pre-service teachers (Chaker et al., 2026) confirms that digital engagement, environmental attitudes, and perceived behavioural control jointly predict environmental citizenship competencies, suggesting that education that integrates these dimensions will produce more robust ecological citizens. Realising this potential in practice, however, requires attending to how digital engagement itself varies across learners: research on collaborative online learning spaces finds that technology adoption patterns differ across generational cohorts, implying that digitally mediated environmental education must be designed with these differences in mind if it is to engage students consistently (Akhter et al., 2026).

Legal frameworks that support environmental education, recognise ecological values in property law and planning, and embed ecological obligations in constitutional provisions do more than regulate behaviour: they shape the cultural and normative environment in which ecological identities are formed. From a positive psychology perspective, this cultural function of law is at least as important as its regulatory function, because it determines the motivational substrate on which behavioural change must rest.

## 5 Conclusions and directions for future research

This article has explored that environmental law, reconceptualised through the lens of positive psychology, is a structural enabler of human flourishing and a catalyst for ecological citizenship. Drawing on the PERMA framework, we have proposed how different domains of environmental legislation activate distinct components of psychological wellbeing: clean environment laws supporting positive emotion; participatory governance enabling engagement; communal ecological responsibilities nurturing relationships; stewardship obligations generating meaning; and institutional effectiveness providing the conditions for civic accomplishment. The reviewed evidence suggests that eco-anxiety represents a growing psychological burden whose mitigation requires not only individual coping strategies but systemic legal responses that restore institutional trust and citizen efficacy.

The flourishing law paradigm proposed in this article is not merely academic. As governments worldwide grapple with the dual challenges of ecological crisis and declining mental health, the recognition that environmental law can serve as both ecological protection and psychological infrastructure offers a practically significant reorientation of legal priorities. It suggests that investments in strong, credible, and participatory environmental governance are simultaneously investments in the psychological welfare of citizens a synergy that may help mobilise the political will necessary for transformative ecological action.

Several directions for future research emerge from this analysis. First, longitudinal studies are needed that directly examine the relationship between changes in environmental legislation and changes in psychological wellbeing indicators at the population level. Second, experimental and quasi-experimental research on the psychological effects of specific participatory governance mechanisms would allow more precise causal claims about the PERMA-activating functions of environmental law. Third, cross-cultural research is needed to examine whether the relationships identified in predominantly Western samples generalise to other ecological and legal contexts, including the distinctive challenges of environmental governance in rapidly developing economies. Fourth, research at the intersection of rights of nature jurisprudence and positive psychology could explore whether legal recognition of non-human subjects of rights generates specific forms of psychological meaning and relational wellbeing.

Fifth and finally, the emerging field of climate litigation psychology examining the psychological motivations and consequences of legal action against governments and corporations for climate inaction represents a particularly promising frontier. As climate litigation proliferates globally, understanding how legal processes can serve both ecological and psychological ends becomes increasingly urgent. The flourishing law paradigm, grounded in the convergence of psychology, ecology, and legal theory, provides a theoretically rich and empirically tractable framework for this work.

## Data Availability

The original contributions presented in the study are included in the article/supplementary material, further inquiries can be directed to the corresponding author.

## References

[ref1] Akhter S. Prasad R. D. Tan M. Iftikhar S. (2026). Beyond tech-fluent generations: investigating cross-generational technology adoption patterns in collaborative online learning spaces. Int. J. Interact. Mob. Technol. 20:75.

[ref2] Albrecht G. (2011). “Chronic environmental change: emerging "psychoterratic" syndromes,” in Climate Change and Human Well-Being, ed. Weissbecker I. (New York, NY: Springer New York), 43–56.

[ref3] Alhendi N. Salameh M. (2023). Environmental pollution crime. J. Environ. Manag. Tour. 14, 176–180. doi: 10.14505/jemt.14.1(65).17

[ref4] Amjad A. I. Javaid S. Saira Akhter S. Khasawneh M. A. S. Al-Kahlan T. B. S. (2026). Influence of digital media use, environmental awareness, and eco-anxiety on university students' mental health. Discov. Psychol. 6:103.

[ref5] Anantharaman M. (2014). Networked ecological citizenship, the new middle classes and the provisioning of sustainable waste management in Bangalore, India. J. Clean. Prod. 63, 173–183. doi: 10.1016/j.jclepro.2013.08.041

[ref6] Atta N. Sharifi A. (2024). A systematic literature review of the relationship between the rule of law and environmental sustainability. Sustain. Dev. 32, 7051–7068. doi: 10.1002/sd.3087

[ref7] Baniamer A. Alhendi N. (2026). Environmental criticism in light of environmental law legislation. Front. Environ. Sci. 14:1807424. doi: 10.3389/fenvs.2026.1807424

[ref8] Bourban M. (2022). Strong sustainability ethics. Environ. Ethics 43, 291–314. doi: 10.5840/enviroethics202211332

[ref9] Bourban M. (2023). Ecological cosmopolitan citizenship. Future Humanit. 1:e5. doi: 10.1002/fhu2.5

[ref10] Brymer E. Freeman E. L. Richardson M. (2024). One health: the psychology of human-nature relationships for planetary and human wellbeing, volume II. Front. Psychol. 15:1386313. doi: 10.3389/fpsyg.2024.1386313 PMC11002199 38596332

[ref11] Butler J. Kern M. L. (2016). The PERMA-profiler: a brief multidimensional measure of flourishing. Int. J. Wellbeing 6, 1–48. doi: 10.5502/ijw.v6i3.526

[ref12] Coffey J. K. Wray-Lake L. Mashek D. Branand B. (2023). PERMA to PERMA+4 building blocks of well-being: a systematic review of the empirical literature. J. Posit. Psychol. 19, 510–529. doi: 10.1080/17439760.2023.2208099, 37339054

[ref9002] Chaker R. de La Hoz-Ruiz J. Miñán Espigares A. Sanchez Mendías J. Hajj-Hassan M. (2026). Environmental citizenship among Spanish pre-service teachers: sustainability awareness and online civic engagement at the University of Granada. Front. Educ. 11:1766746. doi: 10.3389/feduc.2026.1766746

[ref13] Dobson A. (2003). Citizenship and the Environment. Oxford University Press.

[ref14] Doyle R. (2024). Protecting everyday nature. J. Environ. Law 36, 275–302. doi: 10.1093/jel/eqae016

[ref15] Goodman F. R. Disabato D. J. Kashdan T. B. Kauffman S. B. (2018). Measuring well-being: a comparison of subjective well-being and PERMA. J. Posit. Psychol. 13, 321–332. doi: 10.1080/17439760.2017.1388434

[ref9001] Hadjichambis A. C. Paraskeva-Hadjichambi D. (2020). Education for environmental citizenship: The pedagogical approach. Conceptualizing Environmental Citizenship for 21st Century Education. Environmental Discourses in Science Education. vol 4. Cham: Springer. doi: 10.1007/978-3-030-20249-1_15

[ref16] Hadjichambis A. C. Reis P. (2020). “Introduction to the conceptualisation of environmental citizenship for twenty-first-century education,” in Conceptualizing Environmental Citizenship for 21st Century Education, eds. Hadjichambis A. C. Reis P. Paraskeva-Hadjichambi D. Cígel J. Staběrada J. Magntorn-Pugnère D. . (Cham: Springer), 1–18.

[ref18] Huang P. H. (2010). Happiness studies and legal policy. Annu. Rev. Law Soc. Sci. 6, 405–432. doi: 10.1146/annurev.lawsocsci.093008.131615

[ref19] Isham A. Elf P. Jackson T. (2022). Self-transcendent experiences as promoters of ecological wellbeing? Exploration of the evidence and hypotheses to be tested. Front. Psychol. 13:1051478. doi: 10.3389/fpsyg.2022.1051478, 36452396 PMC9701724

[ref20] Kaplan R. Kaplan S. (1989). The Experience of Nature: a Psychological Perspective. Cambridge, England: Cambridge University Press.

[ref21] Kern M. L. Williams P. Spong C. Colla R. Sharma K. Downie A. . (2020). Systems informed positive psychology. J. Posit. Psychol. 15, 705–715. doi: 10.1080/17439760.2019.1639799

[ref23] Nisbet E. K. Zelenski J. M. Murphy S. A. (2011). Happiness is in our nature: exploring nature relatedness as a contributor to subjective well-being. J. Happiness Stud. 12, 303–322. doi: 10.1007/s10902-010-9197-7

[ref24] Orrù L. Mannarini S. (2024). Psychological impact of climate change emergency: an attempt to define eco-anxiety. Front. Psychol. 15:1375803. doi: 10.3389/fpsyg.2024.1375803, 39355285 PMC11442196

[ref25] Pihkala P. (2020). Anxiety and the ecological crisis: an analysis of eco-anxiety and climate anxiety. Sustainability 12:7836. doi: 10.3390/su12197836

[ref26] Prentice M. Jayawickreme E. Fleeson W. (2019). Integrating whole trait theory and self-determination theory. J. Pers. 87, 56–69. doi: 10.1111/jopy.12417, 29999534

[ref27] Rahe M. Jansen P. (2023). A closer look at the relationships between aspects of connectedness and flourishing. Front. Psychol. 14:1137752. doi: 10.3389/fpsyg.2023.1137752, 37063569 PMC10097885

[ref28] Richardson M. Passmore H. A. Lumber R. Thomas R. Hunt A. (2021). Moments, not minutes: the nature-wellbeing relationship. Int. J. Wellbeing 11, 8–22. doi: 10.5502/ijw.v11i1.1267

[ref29] Ryan R. M. Deci E. L. (2000). Self-determination theory and the facilitation of intrinsic motivation, social development, and well-being. Am. Psychol. 55, 68–78. doi: 10.1037/0003-066X.55.1.68, 11392867

[ref30] Seligman M. E. P. (2011). Flourish: a Visionary New Understanding of Happiness and Well-Being. New York, Free Press.

[ref31] Seligman M. E. P. Csikszentmihalyi M. (2000). Positive psychology: an introduction. Am. Psychol. 55, 5–14. doi: 10.1037/0003-066X.55.1.5, 11392865

[ref32] Spano G. Dadvand P. Sanesi G. (2021). Editorial: the benefits of nature-based solutions to psychological health. Front. Psychol. 12:646627. doi: 10.3389/fpsyg.2021.646627, 33613409 PMC7889499

[ref9003] Steele S. L. (2025). Eco-anxiety in higher education professionals: psychological impacts, institutional trust, and policy implications. Eur. J. Investig. Health Psychol. Educ. 16, 6. 10.3390/ejihpe16010006 PMC12839761 41590016

[ref33] Ulrich R. S. Simons R. F. Losito B. D. Fiorito E. Miles M. A. Zelson M. (1991). Stress recovery during exposure to natural and urban environments. J. Environ. Psychol. 11, 201–230. doi: 10.1016/S0272-4944(05)80184-7

[ref34] Vijaikis A. Poškus M. S. (2024). Does being an environmental citizen lead to greater well-being? The self-determination theory approach. SAGE Open 14:21582440241267156. doi: 10.1177/21582440241267156

[ref35] Wexler D. B. (1990). Therapeutic Jurisprudence: the Law as a Therapeutic Agent. Durham, NC: Carolina Academic Press.

[ref36] Wild S. Schulze Heuling L. (2024). Exploring the role of identity in pro-environmental behavior: cultural and educational influences on younger generations. Front. Psychol. 15:1459165. doi: 10.3389/fpsyg.2024.1459165, 39444836 PMC11497401

[ref37] Willis R. L. Brymer E. Sharma-Brymer V. Leach M. (2024). Refocusing on nature: the role of nature reintegration in environmental, mental, and societal wellbeing. Front. Psychol. 15:1351759. doi: 10.3389/fpsyg.2024.1351759, 38476382 PMC10927748

[ref38] Wilson E. O. (1984). Biophilia. Cambridge, MA: Harvard University Press.

[ref39] Winick B. J. (1997). The jurisprudence of therapeutic jurisprudence. Psychol. Public Policy Law 3, 184–206. doi: 10.1037/1076-8971.3.2-3.184

[ref40] Wray-Lake L. Arruda E. H. Schulenberg J. E. (2019). Civic engagement longitudinal change in the transition to adulthood: evidence from the monitoring the future study. Dev. Psychol. 55, 53–65. doi: 10.1037/dev0000619, 30335428 PMC6296892

[ref9004] Zeng Q. Yang Z. Chen Z. Chen P. (2025). The relationships between nature connectedness, nature contact, and positive psychological outcomes: a meta-analysis. J. Environ. Psychol. 105, 102675.

